# Development of Fatal Intestinal Inflammation in MyD88 Deficient Mice Co-infected with Helminth and Bacterial Enteropathogens

**DOI:** 10.1371/journal.pntd.0002987

**Published:** 2014-07-10

**Authors:** Libo Su, Yujuan Qi, Mei Zhang, Meiqian Weng, Xichen Zhang, Chienwen Su, Hai Ning Shi

**Affiliations:** 1 College of Veterinary Medicine, Jilin University, Changchun, Jilin, China; 2 Mucosal Immunology and Biology Research Center, Massachusetts General Hospital and Harvard Medical School, Charlestown, Massachusetts, United States of America; 3 Qinghai University Medical School, Xining, Qinghai, China; Queensland Institute of Medical Research, Australia

## Abstract

Infections with intestinal helminth and bacterial pathogens, such as enteropathogenic *Escherichia coli*, continue to be a major global health threat for children. To determine whether and how an intestinal helminth parasite, *Heligomosomoides polygyrus*, might impact the TLR signaling pathway during the response to a bacterial enteropathogen, MyD88 knockout and wild-type C57BL/6 mice were infected with *H. polygyrus*, the bacterial enteropathogen *Citrobacter rodentium*, or both. We found that MyD88 knockout mice co-infected with *H. polygyrus* and *C. rodentium* developed more severe intestinal inflammation and elevated mortality compared to the wild-type mice. The enhanced susceptibility to *C. rodentium*, intestinal injury and mortality of the co-infected MyD88 knockout mice were found to be associated with markedly reduced intestinal phagocyte recruitment, decreased expression of the chemoattractant KC, and a significant increase in bacterial translocation. Moreover, the increase in bacterial infection and disease severity were found to be correlated with a significant downregulation of antimicrobial peptide expression in the intestinal tissue in co-infected MyD88 knockout mice. Our results suggest that the MyD88 signaling pathway plays a critical role for host defense and survival during helminth and enteric bacterial co-infection.

## Author Summary

Infections with intestinal helminths and enteric bacterial pathogens such as enteropathogenic *Escherichia coli* (EPEC) continue to be major global health problems, especially for children. The ability of the host to control bacterial enteropathogens may be influenced by host immune status and by concurrent infections. Helminth parasites are of particular interest in this context because of their ability to modulate host immune responses, and because their geographic distribution coincides with those parts of the world where infections caused by bacterial enteropathogens are most problematic. In this study, we determined how intestinal helminth infection regulates host innate immunity against bacterial enteropathogens by using a murine co-infection model. This model involves co-infection with the intestinal nematode parasite *Heligmosomoides polygyrus* and the Gram-negative bacterial pathogen *Citrobacter rodentium*, the murine equivalent of EPEC. The infections were carried out in wild-type mice and in mice lacking MyD88, a protein required for signaling by the Toll-like receptors. We found that co-infection with the helminth parasite significantly worsened *Citrobacter*-induced colitis in the MyD88-deficient mice, in association with increased mortality and compromised innate immune responses. Our observations demonstrate an important role for MyD88-dependent and -independent signaling in host survival during helminth and enteric bacterial co-infection.

## Introduction

Infectious gastroenteritis leading to severe diarrhea is a major global health threat, particularly in children. Bacteria such as enteropathogenic *E. coli* (EPEC) are important agents of this disease. The ability of the host to control such bacterial pathogens may be influenced by concurrent infections. Helminth infections, including those caused by soil-transmitted helminths, schistosomiasis and filariasis, form a major group of neglected tropical diseases that affect about one third of the world's population [1]. Co-infection of individual hosts by multiple pathogens can be very commonly observed in natural populations. Although it is well known that helminths induce Th2 polarization of helper T cells, our understanding of the exact mechanism by which these parasites modulate the host's innate defense against concurrently infecting bacterial enteropathogens is incomplete.

Intestinal innate immune cells, such as intestinal epithelial cells, dendritic cells (DCs), macrophages, granulocytes, and innate lymphoid cells (ILCs) provide a first line of defense against pathogens [2]. The innate immune system can be activated through recognition of pathogen-associated molecular patterns (PAMPs) by evolutionarily conserved pattern-recognition receptors (PRRs), such as Toll-like receptors (TLRs). Initiation of signaling cascades induced by TLR engagement requires various adaptor proteins, including myeloid differentiation factor (MyD)88, Toll-IL-1R domain-containing adaptor protein/MyD88 adaptor-like (TIRAP/Mal), and TIR domain-containing adaptor inducing IFNβ (TRIF) and TRIF-related adaptor molecule (TRAM) [3]. MyD88 binds to the TIR domain of the receptors, initiates a signaling cascade, ultimately leading to the activation of proinflammatory genes by transcription factor NF-κB [4]. The MyD88-dependent pathway has been suggested to play important roles in host defense against various mucosal bacterial infections [5]–[7]. MyD88 deficient mice have been shown to develop severe mucosal injury following *C. rodentium* infection [8]. It has been also shown that the inflammation and pathology induced by *C. rodentium* infection are TLR4-dependent [9]. Activation of the Mal/MyD88 pathway leads to increased expression of inflammatory cytokines such as TNF-α, whereas the TRAM/TRIF pathway activates the interferon-regulatory factor-3 transcription factor leading to induction of IFN-β and IFN-inducible genes and NF-κB activation [3].

However, our current understanding of the molecular mechanism by which intestinal helminth parasites influence host immunity to bacterial pathogens is incomplete. Helminth parasites typically induce Th2 and Treg responses. Key roles have been suggested for TLR, Nod-like receptor (NLR) interactions and C-type lectins in the induction of the Th2 response [10]–[12]. To identify signaling cascades responsible for altered immunity to *C. rodentium* in the host with helminth co-infection, we considered the involvement of TLRs, which represent key mediators of innate host defense in the intestine. MyD88 is a common adaptor protein for all TLRs except TLR3. These is evidence to indicate that the absence of MyD88 has no effect on host resistance to the intestinal helminth *Trichuris muris* and that *T. muris*-infected MyD88 knockout mice had an increased Th2 response [13]. However, a recent study showed that MyD88 is required for amplification of the Th2 response to *T. spiralis* infection [14]. These observations indicate that various helminths can have both MyD88-dependent and MyD88-independent effects on immune function. Based on this information, we wished to determine in the current study how loss of MyD88 signaling would impact on host protective immunity and intestinal inflammatory response against enteric bacterial pathogen during helminth co-infection. Our data demonstrate a critical role for the MyD88 signaling pathway in host survival during helminth and enteric bacterial co-infection, and also suggest that helminth infection impairs MyD88-independent responses activated by TLR4.

## Methods

### Mice

Six to 8 week old female MyD88 knockout (were originally generated in the lab of S. Akria, Osaka University, Japan) and C57BL/6 mice (were purchased from The Jackson Laboratory, Bar Harbor, ME) were fed autoclaved food and water and maintained in a specific pathogen-free facility at Massachusetts General Hospital. Animal care was provided in accordance with protocols approved by the Institutional Animal Care and Use Committee of Massachusetts General Hospital.

### *H. polygyrus* infection

*H. polygyrus* were propagated as previously described and stored at 4°C until use [15]. Mice were inoculated orally with 200 third stage larvae. Seven days or 3 weeks (chronic) following parasitic infection, a subset of the *H. polygyrus* infected mice was inoculated with *C. rodentium*.

### *C. rodentium* infection

Mice were orally inoculated with *C. rodentium* (strain DBS100, from ATCC). Bacteria were grown overnight in Luria broth (LB) and resuspended in PBS prior to infecting the mice (0.5 ml/mouse, approximately 5×10^8^ CFU of *C. rodentium)*. To assess the systemic effect of *H. polygyrus* infection, *C. rodentium* infection, and concurrent infection on the host, the body weight and the survival of the infected mice were measured throughout the experimental period. The data presented are pooled data from three independent experiments.

### Histopathological examinations

At necropsy, colonic tissues were collected, frozen in Tissue Tek OCT compound (Miles, Inc., Elkhart, IN) and then stored at 80°C. Then, 5 µm sections were cut on a Leica CM1850 Cryostat (Leica Biosystem) and were stained with hematoxylin and eosin. Intestinal pathology was scored using a modified histology scoring system based on previously published methods [16], [17]. The sections were analyzed without prior knowledge of the type of treatment.

### Immunofluorescence microscopy

Colon tissues were frozen in Tissue-TeK OCT compound. Five-micrometer sections were cut on a cryostat and fixed in ice cold acetone. The sections were then washed and blocked with avidin/biotin agent (Vector Laboratories). To examine intestinal MNP cells, the sections were stained with FITC-labeled anti-mouse Mac-a (CD11b, BD Pharmingen). Sections were analyzed by immunofluorescence microscopy [17].

### Determination of *C. rodentium* translocation

To examine whether co-infection with helminth parasites result in enhanced *C. rodentium* translocation into both the mucosal and the systemic compartments, mice were infected with *H. polygyrus*, and 7 days later they were infected with GFP-expressing *C. rodentium*. Separate mice were infected with *C. rodentium* only or not infected. The mice were sacrificed at 3 and 9 days after bacterial infection. The appearance and distribution of GFP-C. rodentium was examined using immunofluorescence microscopy.

### Quantitative detection of chemokine and antimicrobial peptide expression in colonic tissues

Total RNA was prepared from colon tissues using TRIzol reagent (Invitrogen Life Technologies) following the manufacturer's recommendations and reverse transcribed into cDNA using the Superscript First-Strand Synthesis System (Invitrogen Life Technologies). The cDNA samples were then tested for the expression of chemokine, KC, and antimicrobial peptides, Reg3β, Reg3γ by real-time quantitative RT-PCR using SYBR Green PCR Master Mix (Applied Biosystems) on a StepOne Plus real-time PCR system (Applied Biosystems). Samples were run in triplicate. GAPDH was used as the housekeeping control. The sequences for the sense and antisense primers used to quantify mRNA are: KC: F, CCGAAGTCATAGCCACACTCAA and R, GCAGTCTGTCTTCTTTCTCCGTTAC; RegIIIγ: F, TTCCTGTCCTCCATGATCAAAA and R, CATCCACCTCTGTTGGGTTCA; RegIIIβ: F, ATGGCTCCTACTGCTATGCC and R, GTGTCCTCCAGGCCTCTTT, TNF-α: F, CCCTCACACTCAGATCATCTTCT and R, GCTACGACGTGGGCTACAG, IL-10: F, CCACAAAGCAGCCTTGCA and R, AGTAAGAGCAGGCAGCATAGCA, IL-22: F, TCCGAGGAGTCAGTGCTAAA and R, AGAACGTCTTCCAGGGTGAA, and GAPDH: F, TGGAATCCTGTGGCATCCATGAAAC and R,TAAAACGCAGCTCAGTAACAGTCCG.

### TLR stimulation of macrophages

Peritoneal macrophages were collected from mice infected with *H. polygyrus* (2 weeks post infection) or uninfected control mice. After incubation in complete DMEM for 2 h, non-adherent cells were removed by washing. The adherent cells were incubated in complete DMEM at 37 C with and without LPS (100 ng/ml) or IL-1β (1 ng/ml) for 6 h. The culture supernatants were collected and TNF-α and IL-6 production were measured using ELISA. Total mRNA was prepared from the cultured cells using TRIzol reagent (Invitrogen Life Technology) following the manufacture's recommendations. The cDNA samples were then tested for the expression of KC by real-time RT-PCR.

### Statistical analysis

All of the results are expressed as the mean and the standard error of the mean (SEM). “N” refers to the number of mice used. Statistical differences were determined by using a two-tailed Student t test with StatView software (Abacus Concepts, Berkeley, CA). A P value<0.05 was considered significant.

## Results

### MyD88 is required for survival during enteric bacterial and helminth co-infection

*C. rodentium* infection in most adult mice is self-limiting, with little morbidity and mortality.

**Figure 1:**
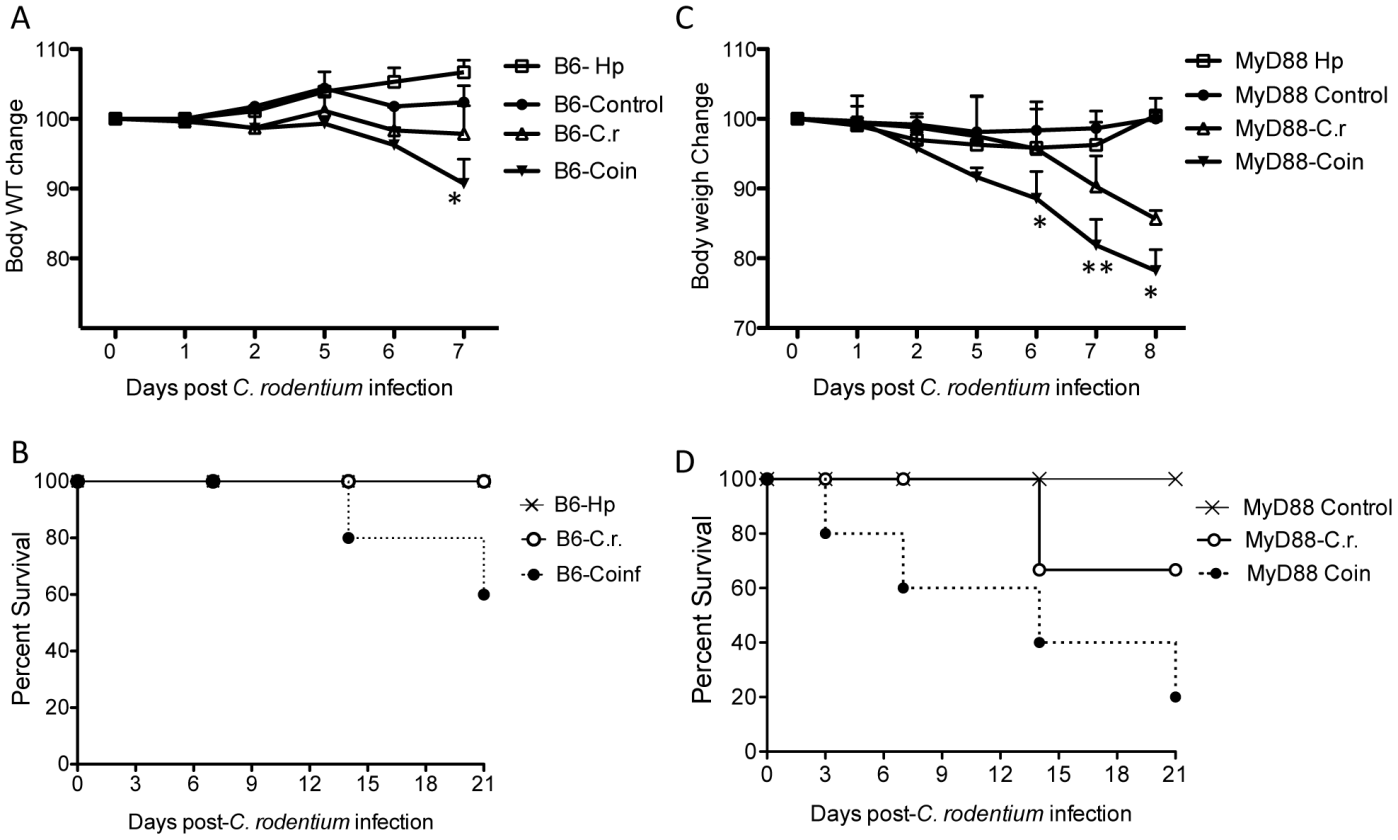
Helminth co-infection exacerbates *C. rodentium-*induced colitis and mortality in MyD88 KO mice. MyD88 knockout and wild-type C57BL/6 mice were infected with *H. polygyrus* (200 L3) and inoculated with *C. rodentium* (5×10^8^ CFU) 7 days later. **A & C:** Body weight changes of wild-type (A) and MyD88 knockout (C) mice that were infected with *C. rodentium*, *H. polygyrus*, both *H. polygyrus* and *C. rodentium*, and normal control mice during the course of the experiment (8 days) are shown. Data shown are pooled from three independent experiments and are expressed as the body weight change as a percentage of the individual mouse initial body weight ± SE (n = 10–15) at each time point. **B &D:** Survival curve in wild-type (B) and MyD88 knockout (D) mice. *H. polygyrus* co-infection results in a significantly increased mortality in MyD88 KO mice.

We previously showed that helminth co-infection in wild-type mice induces exacerbated bacterial colitis via STAT 6-dependent mechanisms involving induction of phenotypic and functional alterations of innate immune cells, such as DCs and macrophages [16], [17]. However, the molecular mechanism by which the parasites influence host protective immunity against bacterial enteropathogens is unclear. To gain further mechanistic insight into how helminth infection impairs host defenses against concurrent infection with an enteric bacterial pathogen, we have used MyD88 knockout mice in this study. MyD88 is a common adaptor protein for all TLRs except TLR3. There is evidence to indicate that various helminths can have both MyD88-dependent and MyD88-independent effects on immune function [13], [14]. In the current study we wished to find out if the exacerbating effect of *H. polygyrus* on *Citrobacter* infection required MyD88. MyD88 knockout and wild-type mice were infected with *H. polygyrus* and inoculated with *C. rodentium* 7 days later. As expected, the wild type C57BL/6 mice had signs of *Citrobacter* associated disease, such as soft stool, a hunched posture, disturbed body hair, and body weight loss during the infection, but recovered by 2 to 3 weeks post-infection, which is similar to the observations that we reported previously in BALB/c mice [18]. Co-infection with helminth parasite in these mice results in the development of increased disease severity compared to *C. rodentium* infection alone group (Figure 1 A and B). In contrast, mice with defects in MyD88 signaling develop more severe bacterial infection and bacterial induced disease, as evidenced by results showing gastrointestinal tract bleeding, and development of anal prolapse after bacterial inoculation and higher mortality rate. Such pathology was not observed with the wild-type mice infected with *C. rodentium* alone (data not shown). Co-infection of MyD88 knockout mice with *H. polygyrus* results in the development of more severe disease, more significant and prolonged body weight loss during the course of the experiment (Figure 1C). Furthermore, co-infection of MyD88 knockout mice results in a significant increase in mortality that occurred as early as 3 days after *C. rodentium* infection compared to MyD88 knockout mice with *C. rodentium* infection alone (Figure 1D). These observations demonstrate that mice with a defect in MyD88 signaling pathway are more susceptible to enteric bacterial pathogen and that concurrent infection with intestinal nematode parasite results in the exacerbation of enteric bacterial infection and bacterial induced disease. Therefore, MyD88-dependent mechanisms are required to protect against *Citrobacter* infection, particularly in the context of *H. polygyrus* co-infection.

### Helminth co-infection exacerbates bacteria-induced mucosal injury in MyD88 knockout mice

**Figure 2:**
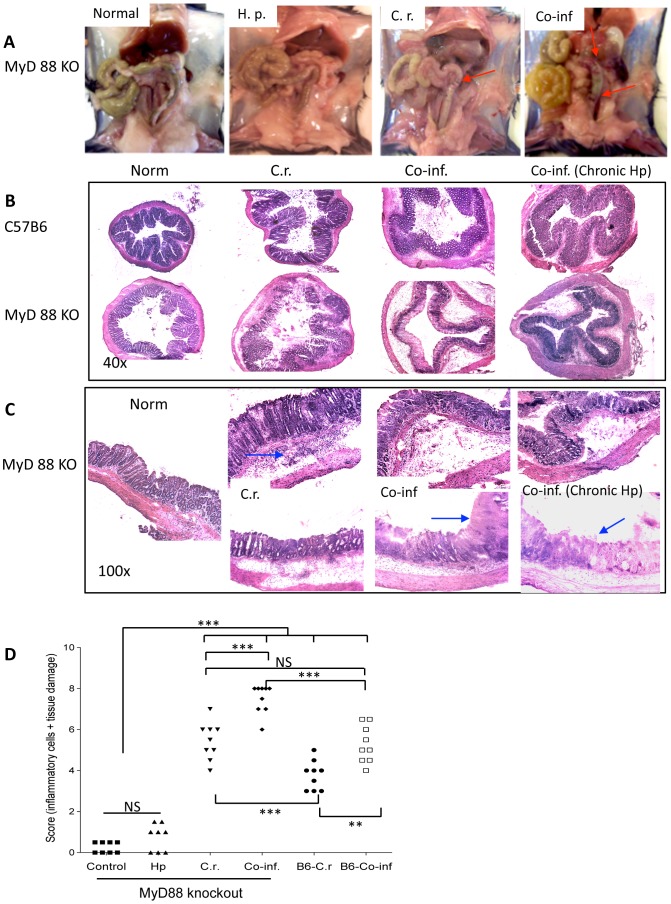
Helminth co-infection exacerbates *C. rodentium-*induced colitis and intestinal injury in MyD88 knockout mice. **A**. Macroscopic examination of colon tissues of MyD88 knockout mice from different treatment groups. Arrows indicate intestinal bleeding. B. Colon tissues were removed from uninfected mice or from mice infected with *H. polygyrus*, *C. rodentium*, or both two weeks after bacterial infection, frozen in Tissue Tek OCT compound, and the sections were stained with hematoxylin and eosin. **B**: Magnification, ×40 and **C**: Magnification, ×100. Duplicate samples are presented from co-infected and *C. rodentium*-infected MyD88 knockout mice. Co-inf (chronic): C57BL/6 and MyD88 knockout mice were pre-infected with *H. polygyrus* for 3 weeks and then co-infected with *C. rodentium*. Arrows indicate cellular infiltration and loss of intestinal architecture. **D**. Histopathological score of colonic inflammation in mice infected with *C. rodentium* or both. The scores were assessed by determination of infiltration of inflammatory cells (score range, 0 to 4), together with the evaluation of cecal tissue damage (score range, 0 to 4). The data shown are pooled from three independent experiments with total (n = 9 to 12 per group). *** p<0.001, **p<0.005.

*C. rodentium* infection induces similar intestinal pathological changes to that seen in many murine models of colitis, including thickening of the wall of the colon, colonic crypt hyperplasia, goblet cell depletion, and mucosal erosion. We determined the role of MyD88 signaling in controlling intestinal mucosal immune and inflammatory responses against intestinal bacterial pathogen during concurrent helminth infection. At necropsy, the colons from the different groups (*C. rodentium* alone, *H. polygyrus* alone, co-infection, and non-infected controls) of wild type C57BL6 and MyD88 knockout mice were examined both macroscopically and microscopically. In contrast to wild-type mice, MyD88 knockout mice that were infected with *C. rodentium* developed more severe tissue damage including thickening and shortening of the colons with bleeding (Figure 2A). Our results further show that an intestinal helminth co-infection results in the development of more pronounced damage in colon of MyD88 knockout mice than that observed in MyD88 knockout infected with *C. rodentium* alone or in WT mice with *C. rodentium* alone or co-infection (Figure 2A).

We next evaluated bacterial induced colonic pathology in mice from various groups microscopically. Histological examination of the colon of *C. rodentium*-infected wild type C57B/6 mice (at 7 day after *C. rodentium*-infection) showed typical pathological changes associated with this bacterial pathogen, including colonic epithelial cell hyperplasia, crypt elongation, disruption of epithelial architecture and marked infiltration by PMNs (Figure 2B). In contrast, microscopic analysis of colonic tissues of MyD88 knockout mice at 7 days post *C. rodentium* infection showed a more severe pathology compared to *Citrobacter*-infected wild-type mice, including colonic edema, disruption of epithelial architecture, severe ulcerations in colon as well as cecum (Figure 2B and C). A marked cellular infiltration of the colonic lamina propria and submucosa was also evidenced in *C. rodentium*-infected MyD88 knockout mice (Figure 2B and C).

Histological examination of colonic tissue of co-infected mice shows that a concurrent intestinal helminth infection exacerbates bacteria-induced mucosal injury in both B6 and MyD88 knockout mice. In comparison to wild-type C57BL/6 mice, MyD88 knockout mice that were co-infected with helminth developed more severe intestinal pathology, including colonic crypt rupture, disruption of normal alignment of colonic epithelial cells in (Figure 2B). Moreover, in MyD88 knockout co-infected mice, the severe edema of the gut wall, the stunted and/or complete destructed crypts and ulcerations were evident (Figure 2B and C). In fact, in some of the co-infected MyD88 knockout mice the colon tissues lost the crypt architecture completely (Figure 2B and C). The pathology scores for inflammation and intestinal damage were significantly higher in co-infected hosts (both MyD88 knockout and wild-type C57BL/6) compared to their counterparts that were infected with *C. rodentium* alone (Figure 2D). The pathology scores for co-infected MyD88 knockout mice were found to be significantly higher than those of the wild-type mice with helminth-coinfection (Figure 2D). To further examine the effect of concurrent helminth infection on host response to enteric bacterial infection in MyD88 knockout mice, a chronic helminth infection protocol was also utilized in our study. Mice were infected with *H. polygyrus* for 3 weeks and then inoculated with *C. rodentium*. Histological analysis of colonic tissue of co-infected MyD88 knockout mice shows that a chronic intestinal nematode infection also results in the development of exacerbated intestinal injury (Figure 2B). These results, therefore, demonstrate that loss of MyD88 signaling results in a dramatic increase in host susceptibility to non-invasive enteric bacterial pathogen and promotes bacterial induced mucosal injury during concurrent intestinal helminth infection.

### Helminth infection results in increased disseminated bacterial infection

**Figure 3:**
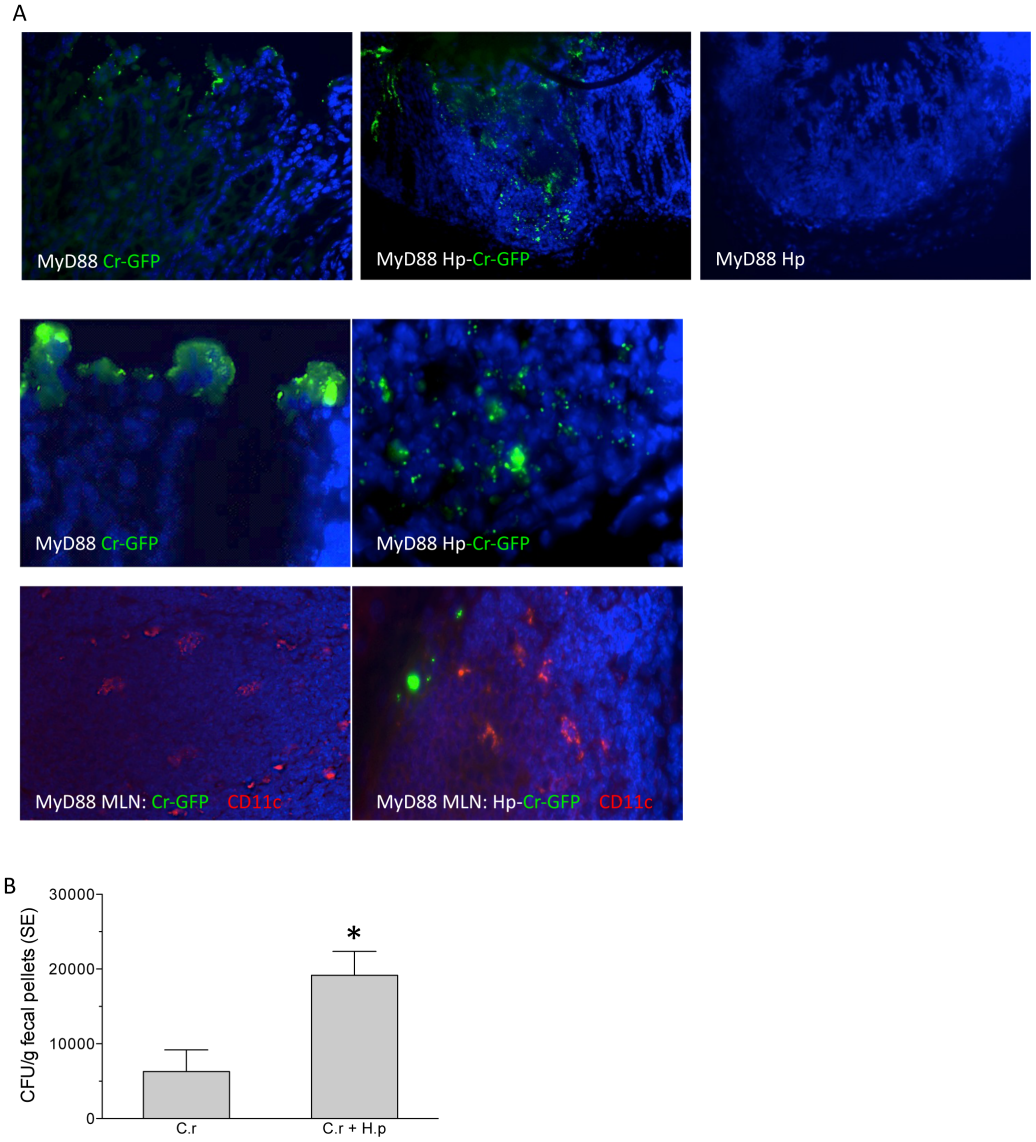
Helminth infection results in enhanced *C. rodentium* translocation in colonic tissue of MyD88 KO mice. **A.** Immunofluorescence microscopic analysis of bacterial distribution and localization in the colonic tissues at day 5 post *C. rodentium* infection. Most of the bacteria were detected in the intestinal epithelial surface in MyD88 knockout mice with bacterial infection alone. An increased bacterial translocation was detected in MyD88 knockout mice with helminth-coinfection. Green: GFP-*C. rodentium*, Blue: DAPI. Red: CD11c in MLN. B. Numbers of bacteria recovered from fecal samples of *C. rodentium*-infected and co-infected MyD88 knockout mice at 7 days post-infection. The data shown are represented as the mean ± the SEM (n = 6 to 7 mice). *p<0.05.

To gain mechanistic insight into how helminth infection impairs host defenses against concurrent infection with a bacterial enteropathogen, MyD88 knockout mice (with and without helminth-coinfection) were infected with GFP-expressing *C. rodentium*. Immunofluorescence microscopic analysis of bacterial distribution and localization in the colonic tissues revealed that at day 5 post *C. rodentium* infection, most of the bacteria were found on the intestinal epithelial surface in the bacterial infection alone group (Figure 3). In contrast, a marked increase in bacterial translocation was detected in MyD88 knockout mice with helminth-coinfection, evidenced by detection of significantly increased distribution of GFP-*C. rodentium* in the LP of colon tissue (Figure 3A). An increase in bacterial translocation was further demonstrated by the observation of increased bacterial detection (Figure 3) and recovery in MLN in co-infected mice as compared to mice infected with *C. rodentium* alone (data not shown). Moreover, examination of fecal bacterial output suggests that helminth co-infection results in significantly increased bacterial shedding in MyD88 knockout mice (Figure 3B). These observations, therefore, suggest that helminth infection of MyD88 knockout mice results in pronounced impairment of intestinal epithelial barrier function and protection (Figure 2), contributing to increased bacterial replication and translocation.

### MyD88 deficiency results in reduced intestinal phagocytic cell recruitment in mice co-infected with enteric helminth and bacterial pathogens

**Figure 4:**
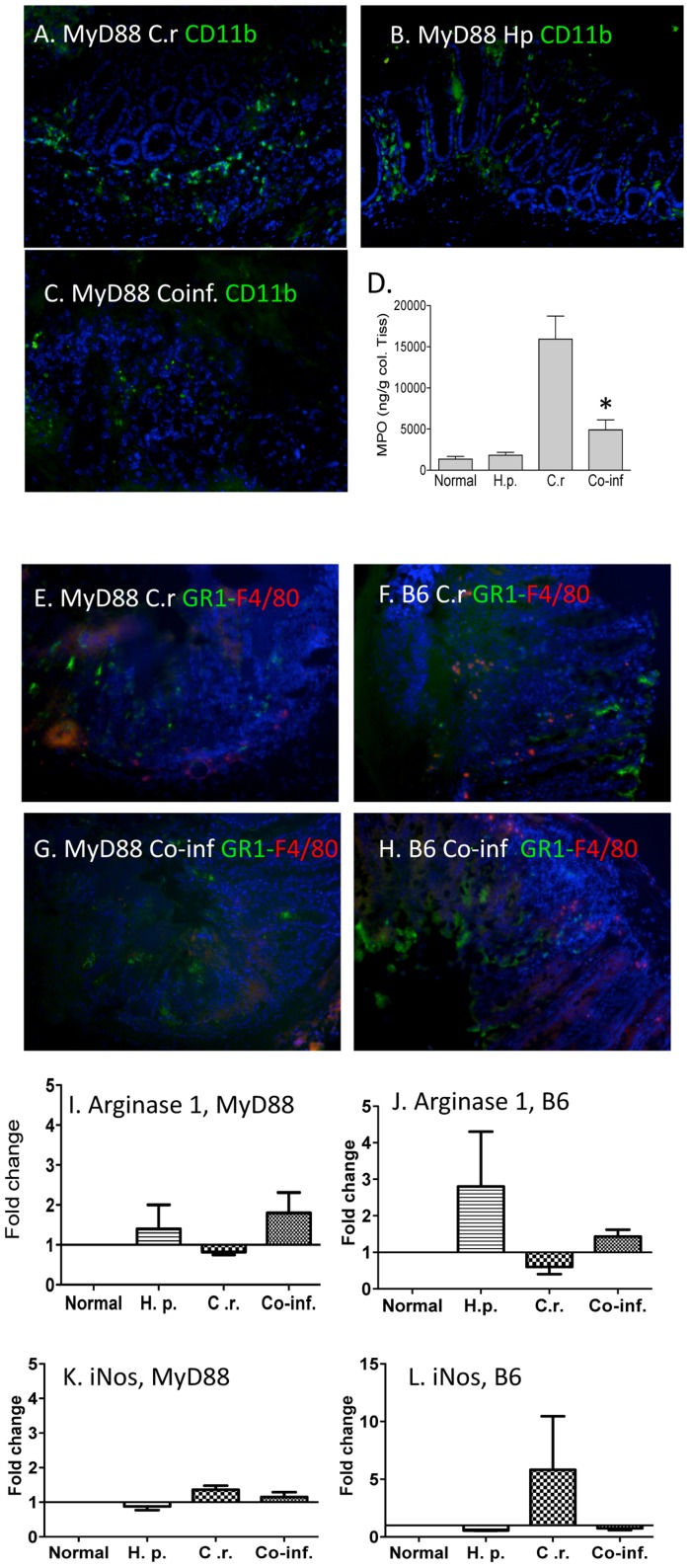
Helminth co-infection results in decreased number of CD11b^+^ cells and antimicrobial peptide expression in colonic tissue. **A–C**, MyD88 knockout mice were infected with *H. polygyrus* and inoculated with *C. rodentium* orally 7 days later. Mice infected with *C. rodentium* (C.r) (**A**), *H. polygyrus* (Hp) (B) and both (Co-inf) (**C**) were sacrificed. Histological sections of the colon were stained with anti-CD11b-FITC (green) and DAPI (blue), and analyzed by immunofluorescence microscopy. Magnification, ×100. **D**, Colonic tissue MPO level was determined by ELISA. **E–H**. Colon tissues were collected from *C. rodentium*-infected (**E**: MyD88 knockout; **F**: C57BL/B6) and co-infected (**G**: MyD88 knockout and **H**: C57BL/B6) mice and stained with anti-GR1-FITC (green), anti-F4/80 (red) and DAPI (blue), and analyzed by immunofluorescence microscopy. All images were digitized and cropped in Adobe Photoshop LE 5.0 (Adobe Systems). **I**–**L**:Colon tissues were collected from normal control, *C. rodentium*-infected, *H. polygyrus* infected, and co-infected wild-type C57BL/6 and MyD88 knockout mice. The expression of Arginase 1, a marker for alternatively active macrophages, was determined in MyD88 knockout mice (**I**) and B6 mice (**J**) using quantitative RT-PCR. iNOS expression was determined in MyD88 knockout mice (**K**) and B6 mice (**L**). Values are the fold increase compared with baseline obtained from uninfected mice. The data shown are the mean ± the SEM (n = 3–5 mice/group) from one of three experiments performed showing similar results. *p<0.05.

To elucidate the cellular basis for the significant impairment of host protective immunity against *C. rodentium* infection in helminth co-infected host, we examined the frequency and distribution of innate immune cells that are known to play an important role in mucosal defense against bacterial infection, specifically, macrophages and neutrophils, in the colonic tissue of MyD88 knockout mice with *C. rodentium* infection. Immunofluorescence microscopy study revealed that LP CD11b^+^ cells (macrophages) were readily detectable in the colonic tissues of MyD88 knockout mice that were infected with *C. rodentium* or helminth (Figure 4A & B). Therefore, the CD11b^+^ cells are being recruited in a MyD88-independent fashion. In sharp contrast, a marked reduction in the frequency of colonic CD11b^+^ cells in colonic LP was detected in MyD88 knockout mice that were co-infected with helminth and the bacterial pathogens (Figure 4C). Therefore, the helminth infection is impairing this MyD88-independent recruitment of CD11b^+^ cells. To further test the possibility that defects in MyD88 pathway and concurrent helminth infection may influence innate immune cells, contributing to impaired host protection, colonic myeloperoxidase (MPO) levels, indicative of granulocyte/neutrophils infiltration, was determined. Our results show that *C. rodentium* infection results in an elevation of colonic MPO levels, which were found to be significantly suppressed in helminth-coinfected host (Figure 4D). Although CD11b has been widely used as a pan-macrophage marker, CD11b is also expressed on other cell types, including neutrophils. The observations of the reduced frequency of colonic CD11b^+^ cells (Figure 4C) are in line with immunoflourescence microscopy experiments showing a reduced frequency of both GR1^+^ neutrophils and F4/80^+^ macrophages in the colonic LP of MyD88 knockout mice with helminth co-infection (Figure 4G and H) compared to MyD88 knockout mice with *C. rodentium* alone as well as co-infected wild-type C57BL/6 mice (Figure 4E–H). These results, therefore, suggest that *Citrobacter* induces neutrophil recruitment and macrophage infiltration via a MyD88-independent mechanism and that *H. polygyrus* infection impairs this mechanism. Further real time RT-PCR analysis of colonic tissue revealed increased expression of Arginase-1, a marker for alternatively activated macrophages [17], in both MyD88 knockout as well as C57BL/6 mice (Figure 4I and J), demonstrating that *H. polygyrus* induces the development of alternatively activated macrophages. In addition, we observed that *C. rodentium*-infection induces iNOS expression in C57BL/6, but not in MyD88 knockout mice, suggesting the effect of MyD88 deficiency on the development of M1 macrophages.

**Figure 5:**
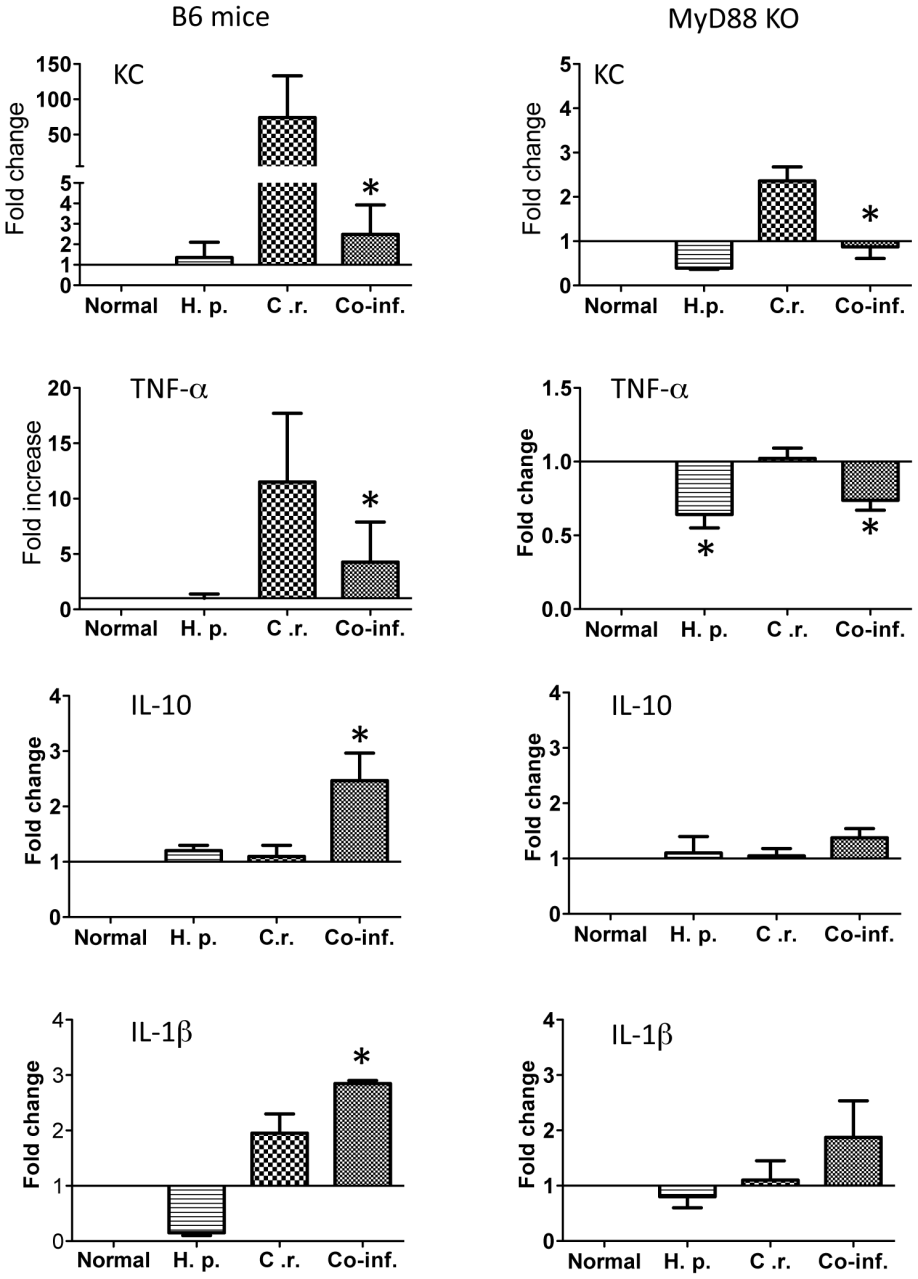
Co-infection with *H. polygyrus* results in dysregulation of *Citrobacter-*induced colonic cytokines. Colon tissues were collected from normal control, *C. rodentium*-infected, *H. polygyrus* infected, and co-infected wild-type C57BL/6 and MyD88 knockout mice. Total RNA was isolated. The expression of KC, TNF-α, IL-10 and IL1β was determined using quantitative RT-PCR. Values are the fold increase compared with baseline obtained from uninfected mice. The data shown are the mean ± the SEM (n = 3–5 mice/group) from one of three experiments performed showing similar results. *p<0.05.

Moreover, in the current study we determined the expression of one of the major chemoattractants for recruiting neutrophils: KC (CXCL1) in the colon tissue in both wild-type and MyD88 knockout mice. Our real-time quantitative RT-PCR analysis showed that *C. rodentium* infection induced a significant up-regulation of the expression of this chemokine (Figure 5) in wild-type mice. In contrast, the KC expression level is much lower in MyD88 knockout mice (Figure 5). Therefore, MyD88-dependent signals play an important role in *Citrobacter*-induced KC expression, although there is a small residual MyD88-independent mechanism that leads to KC up-regulation. Our results further show that *H. polygyrus* co-infection suppresses bacterial induced KC response (Figure 5) in both wild-type and MyD88 knockout mice and that, in fact, such response is completely abolished in MyD88 knockout mice (Figure 5). The fact that *H. polygyrus* also suppresses *Citrobacter*-induced KC expression in MyD88 KO mice suggests that the helminth infection is acting on a MyD88-independent mechanism of KC expression. The results suggest that co-infection with intestinal nematode parasite negatively regulates intestinal recruitment of mononuclear phagocytes and neutrophils, which is correlated with significant reduction of colonic chemokine expression in mice, particularly those lacking MyD88 signaling. Further real time quantitative RT-PCR analysis of colonic tissues showed the helminth-infection induced down-regulation of TNF-α expression in both wild-type and MyD88 knockout mice with *H. polygyrus*-infection, suggesting the impact of helminth infection on TNF-α expression is MyD88-independent. Our data further showed up-regulation of IL-10 in co-infected WT mice (Figure 5), indicating that helminth infection acts on a MyD88-dependent mechanism of colonic IL-10 response. IL-1β expression detected in co-infected mice appeared to be at least partially MyD88-independent (Figure 5).

### Helminth infection negatively regulates TLR-signaling

**Figure 6:**
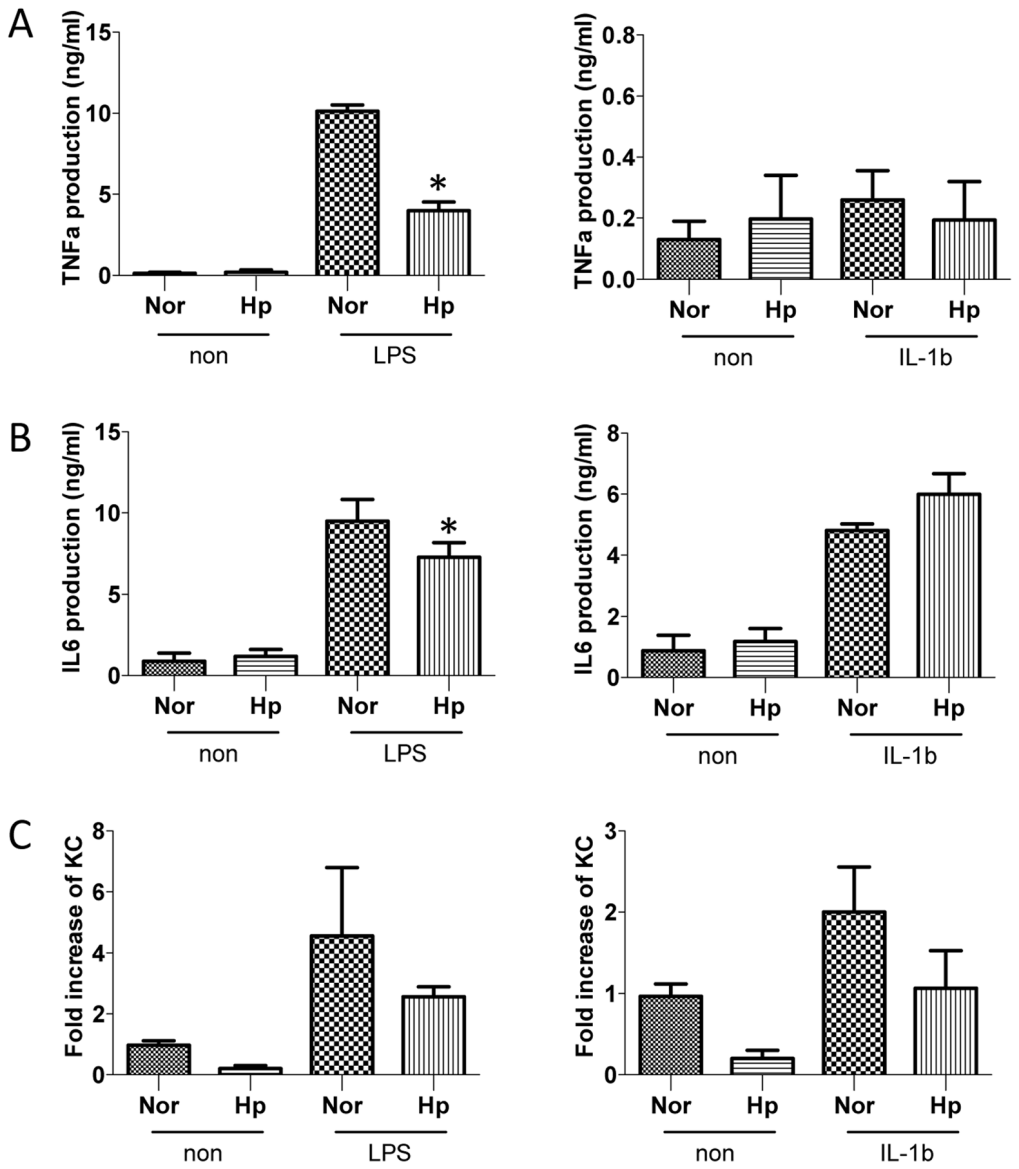
*H. polygyrus*-infection induces the inhibition of LPS-, but not IL-1β-, induced pro-inflammatory cytokine production. The peritoneal macrophages were collected from normal and helminth-infected mice and re-stimulated the cells *in vitro* with LPS (100 ng/ml) or IL-1β (1 ng/ml) for 1 or 6 h. Culture supernatants were collected (at 6 h). TNF-α (**A**) and IL-6 (**B**) production was measured by Cytokine ELISA. KC expression (**C**) (at 1 h) was determined by Real time RT-PCR. *p<0.05, n = 5–10 mice per group.

Recent evidence suggests that TLR signaling in tissue macrophages directly controls the synthesis of neutrophil-attracting chemokines that are essential for the earliest recruitment step in the innate immune response to microbial challenge [19]. To directly test the impact of helminth infection on TLR signaling, we collected the peritoneal macrophages from normal and helminth-infected mice and stimulated the cells with LPS (100 ng/ml) or IL-1β (1 ng/ml) for 6 h. Cytokine ELISA data showed that helminth infection significantly inhibited LPS-, but not IL-1β-, induced TNF-α and IL-6 production (Figure 6 A and B), suggesting that helminth infection specifically inhibits the TLR4 signaling pathway. TLR4 pathway activates distinct signal transduction pathways involving Mal/MyD88 and TRAM/TRIF signaling adaptors, leading to increased expression of inflammatory cytokines such as TNF-α [3]. Since the MyD88-mediated pathway plays a key role in IL-1R signaling [3], unimpaired IL-1β–induced TNF-α and IL-6 production by macrophages from helminth-infected host suggests that the effect of the helminth infection is not on MyD88-dependent signals, but is likely to be on the TRAM/TRIF pathway. To further determine the impact of helminth-infection on TLR signaling, we treated peritoneal macrophages (from normal and helminth-infected mice) with LPS (100 ng/ml) or IL-1β (1 ng/ml) for 1 h and found that the expression of the neutrophil chemoattractant KC was reduced in macrophages isolated from infected mice after LPS-stimulation (Figure 6C).

### Helminth-coinfection results in an inhibition of the expression of antimicrobial peptide in colonic tissue

**Figure 7:**
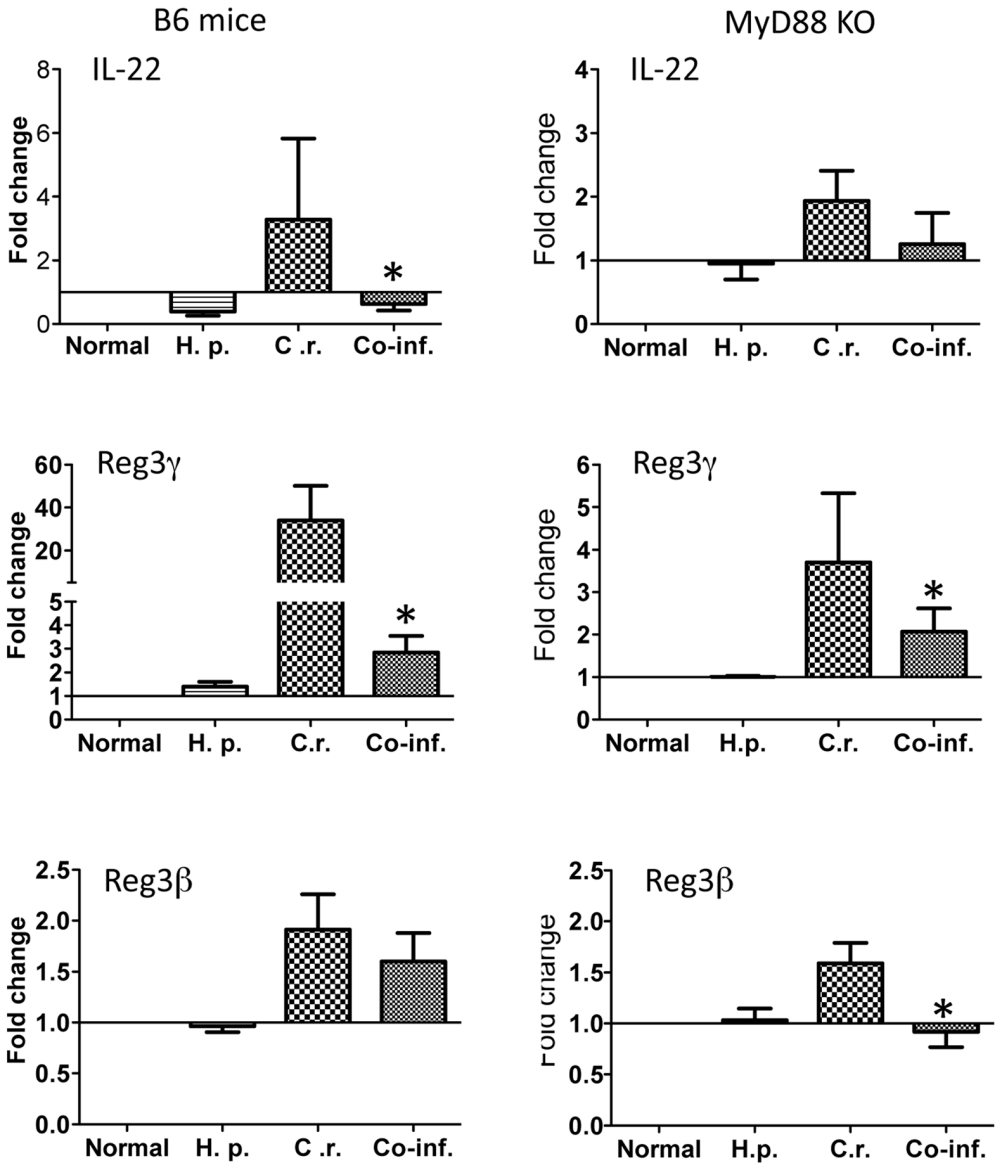
Co-infection with *H. polygyrus* results in down-regulation of *C. rodentium-*induced colonic anti-microbial peptide expression. Colon tissues were collected from control, *C. rodentium*-infected, *H. polygyrus* infected, and co-infected MyD88 knockout and wild-type mice. Total RNA was isolated. IL-22, Reg3γ and Reg3β expression was determined using quantitative RT-PCR. Values are the fold increase compared with baseline obtained from uninfected mice. The data shown are the mean ± the SEM (n = 3–5 mice/group) from one of three experiments performed showing similar results. *p<0.05.

Innate mucosal antimicrobial peptides have been shown to be important for *C. rodentium* infection [20], [21]. We determined the impact of MyD88 defect and helminth-coinfection on host mucosal antimicrobial peptide response by examining RNA expression of Regenerating Islet-Derived 3 (Reg3)γ and Reg3β, both of which have been shown to be induced by IL-17 and IL-22, and to play a protective role in *C. rodentium* infection [21], [22]. Real time RT-PCR of colonic tissue showed a reduction of IL-22 expression in co-infected host (Figure 7A and B). Our results further show that colonic tissues obtained from *C. rodentium*-infected mice (both wild-type and MyD88 knockout) display a significantly elevated level of Reg3γ and Reg3β (Figure 7). However, *C. rodentium*-associated Reg3γ expression is much more pronounced in the colon of wild-type mice compared to that in MyD88 knockout mice (Figure 7), suggesting a role for MyD88 signaling for a maximum induction of this antimicrobial peptide in response to *C. rodentium* infection. Moreover, our results show that helminth-coinfection results in a significant inhibition of *C. rodentium*-induced antimicrobial peptide Reg3β expression in the colon of MyD88 knockout mice (Figure 7).

In the current study we also observed that *H. polygyrus*-infection induced normal development of Th2 responses in MyD88 knockout mice compared to C57BL/6 mice. This was evidenced by the detection of elevated levels of serum IgG1 in MyD88 knockout (305±40 µg/ml) and C57BL/6 mice (260±35 µg/ml) after *H. polygyrus* infection (2 weeks post-infection) (P>0.05), comparing to 180±20 µg/ml and 140±30 µg/ml in un-infected MyD88 knockout and C57BL/6 mice, respectively.

## Discussion

*C. rodentium* is an attaching and effacing pathogen in mice and has been used as the animal model for studying the pathogenesis of EPEC and enterohemorrhagic *E. coli* and for investigating the host response to enteric pathogens and the resulting immunopathology [23], [24]. Studies with various gene knockout mice deficient in T and B cells have indicated an essential and protective role of T and B cells in host defense [25], [26]. It has also been well recognized that innate immunity and the epithelial barrier play a fundamental role in intestinal infection and inflammation. Activation of innate immunity via TLRs leads to an immediate response to infection and can profoundly influence the development of an adaptive immune response. The MyD88-dependent pathway has been suggested to contribute to host defense against various mucosal bacterial infections [5]–[7], including the non-invasive enteric bacterial pathogen, *C. rodentium* infection [8], [27]. In this current study, we have examined the influence of intestinal helminth infection on host innate immunity and the pathogenesis of *C. rodentium* infection using MyD88 knockout mice. Our data show that co-infection with helminth parasite results in the development of exacerbated *C. rodentium*-induced intestinal inflammation and tissue injury and fatal colitis in mice that lost MyD88-dependent signaling pathway. The enhanced susceptibility to *C. rodentium*-infection and exacerbated intestinal injury in the co-infected mice was found to be associated with diminished innate immune responses to *C. rodentium* infection, as evidenced by the inhibition of anti-microbial peptide expression, the reduction of *C. rodentium*-induced expression of KC, and impaired neutrophil recruitment in the intestine (Figure 4, 5 and 7).

The exacerbated bacteria-induced intestinal injury and the development of lethal colitis in MyD88 knockout mice that are co-infected with helminth parasites may be the consequences of the alterations in colonic epithelial barrier function and intestinal innate antibacterial defense mechanism associated with loss of MyD88 signaling pathway and concurrent helminth infection. Following oral infection, *C. rodentium* colonizes the colon. However, we observed increased bacterial numbers in colonic LP in MyD88 knockout mice that are co-infected with helminth parasite, suggesting a role for MyD88 signaling and/or helminth infection in regulating intestinal mucosal barrier function. It was reported previously that MyD88 knockout mice could not promote epithelial cell turnover and repair, leading to deep bacterial invasion of colonic crypts, and intestinal barrier dysfunction [8]. A previous study also showed that MyD88 knockout mice treated with dextran sodium sulfate (DSS, in drinking water) developed more severe intestinal tissue damage than control mice, and that these mice also frequently grew Gram-negative bacteria in the MLN [28], [29], and developed more severe disease and mortality [27]. Our recent study demonstrated that *H. polygyrus* infection resulted in a STAT6-dependent increase in permeability in the colon [30], which may facilitate the movement of luminal contents across the mucosa. It may be speculated that the insults from both Myd88-deficiency and concurrent helminth infection on the intestinal epithelium may synergize and lead to more pronounced disruption of mucosal epithelium, contributing to increased dissemination of intestinal bacteria in MyD88 knockout mice that are co-infected with both pathogens.

Recent evidence suggests a protective role for innate mucosal antimicrobial peptides, such as IL22-dependent Reg3γ and Reg3β in *C. rodentium* infection [21], [22]. Our results from the current study show that IL-22 expression as well as these innate antibacterial responses are found to be significantly inhibited in helminth-coinfected MyD88 knockout mice (Figure 7). We observe that *C. rodentium* infection induces upregulation of antimicrobial peptide expression Reg3γ expression (Figure 5) in both wild-type and MyD88 knockout mice and that this response was severely impaired in MyD88 knockout mice in comparison to wild-type mice, supporting a role for MyD88 signaling in the induction of mucosal antimicrobial peptide response [31]. This is supported by the results from a study that addressed the role for epithelial MyD88 in maintaining intestinal homeostasis and showed that transgenic expression of MyD88 by Paneth cells in MyD88 knockout mice induced antimicrobial peptide response upon sensing of commensal bacterial and prevents bacterial translocation [32]. Interestingly however, our data show that *C. rodentium*-induced Reg3β expression is completely abolished in co-infected MyD88 knockout mice, further suggesting a negative regulatory effect of the parasite on innate mucosal antimicrobial peptide response. In addition to inhibition of antimicrobial peptide expression, both MyD88 deficiency and helminth infection have been shown to impact on autophagy-mediated killing of bacterial pathogens, which may be another mechanism contributing to impaired defense against invading bacterial enteropathogens. A recent study has shown that MyD88 signaling pathway is necessary for the activation of autophagy of intestinal epithelial cells following *Salmonella* infection and that defects in this signaling pathway contribute to impaired protection against bacterial invasion [33]. Our recent study has demonstrated that helminth-infection impairs autophagy-mediated killing of bacterial enteropathogens in BALB/c mice [34]. The results from this current study provide evidence to demonstrate that *H. polygyrus* infection-induced development of alternatively activated macrophages is MyD88 independent (Figure 4 I and J), which may contribute to impaired control of bacterial infection [34]. Taken together, these results demonstrate a role for MyD88 signaling in regulating host innate immune response and in facilitating host survival during concurrent infection with enteric bacterial and helminth pathogens. They also extend our current understanding of the immunoregulatory mechanism of helminth infection to include a down-regulation of innate immunity by helminth infection and/or helminth-induced adaptive immune response.

Intestinal phagocytes are important cell types in the intestinal mucosa that are involved in recognition of bacteria through the TLR-MyD88 pathway [35], [36]. Recruitment of MNP plays a vital role in controlling infections. Deficiency in neutrophils resulted in increased susceptibility to bacterial and fungal infections [37]. These cells are able to deploy a number of key anti-microbial mechanisms, including phagocytosis, the generation of reactive oxygen species, and the release of neutrophil extracellular traps [37]–[39]. Our data showed that neutrophil recruitment is reduced in helminth co-infected mice. This is in line with the report showing helminth-induced IL-4 and IL-13 responses suppressed neutrophil infiltration and pro-inflammatory cytokine responses during *Schistosomiasis japonica* infection in mice [38]. Recent evidence showed MyD88-dependent recruitment of MNP to lungs is required for protection against *B. mallei* infection [40]. Further elucidation of the potential mechanism responsible for the observed reduced colonic phagocytic cell infiltration reveals that the induction of bacterial-induced KC expression, one of the major chemoattractants responsible for recruiting neutrophil, is MyD88 dependent (Figure 5) and that helminth co-infection completely abolished such response in MyD88 knockout mice (Figure 5). This is in line with a previous report showing that KC (as well as MIP-2) are produced by signaling through MyD88 in macrophages, and that MIP-2 can be also synthesized through the TRIF adaptor protein [19]. In addition, a markedly reduced distribution and frequency of colonic CD11b^+^ macrophages in MyD88 knockout during bacterial infection as well as co-infection may contribute to reduced production of KC and MIP-2 [19]. The impaired recruitment of phagocytic cells is likely to be an important factor contributing to observed impaired host defense in mice co-infected with bacterial enteropathogen and intestinal helminth parasite.

Our study raises some important questions about the role of MyD88 in intestinal pathophysiology in the context of helminth-coinfection. In the current study, we observed that helminth co-infection in MyD88 knockout mice results in the development of fatal colitis, yet there was no clear evidence showing an increase in proinflammatory cytokine production in the infected colon tissue (Figure 5). It is unclear what specific factors or molecules are involved in causing the exacerbation of the intestinal inflammation in MyD88 knockout mice that are co-infected with helminth parasite. The influence is clearly helminth-dependent and MyD88 independent. It was reported that *C. rodentium* infection of FVB mice, an immunocompetent strain, induces the development of high mortality and severe colitis that is associated with a level of proinflammatory gene expression comparable to Swiss Webster mice, which had minimal inflammation in the colon [41]. However, *C. rodentium*-associated mortality and severe colitis in FVB mice can be prevented by fluid therapy [41]. A previous study tested the possibility that MyD88-dependent signaling protects from *C. rodentium* through blocking epithelial apoptosis and found no significant increase in apoptosis in colonic tissue from MyD88 knockout animals compared with WT animals, suggesting that necrosis may be responsible for the exacerbated pathology detected in infected MyD88 knockout mice [27]. It is likely that helminth co-infection may accelerate such response through undefined mechanism(s), contributing to the development of fatal colitis in MyD88 knockout mice. To address this question, we stimulated the peritoneal macrophages (isolated from normal or helminth-infected WT mice) with LPS or IL-1β and found defective macrophage response to the TLR4 ligand stimulation in TNF-α and IL-6 secretion in cells isolated from helminth-infected host (Figure 6). TLR4 induces two distinct signaling pathways dependent on 2 distinct pairs of adaptor proteins – Mal and MyD88 and TRAM and TRIF [3]. The data from this current study provides evidence to indicate the possibility that in MyD88 knockout mice helminth-infection may exert its impact on the TRAM-TRIF signaling pathway, contributing to the exacerbated tissue damage and enhanced fatality.

In summary, we report here that MyD88 is a critical molecule required for effective innate immunity against the enteric bacterial pathogen *C. rodentium*. Significantly, the results reported in this article have demonstrated a role for MyD88 signaling in host survival during concurrent infection with intestinal parasitic and bacterial pathogens. The major immune defects in co-infected MyD88 knockout host appear to be related to the disrupted intestinal epithelial barrier function and diminished intestinal phagocytic cells. The results from the current study suggest that in helminth co-infected MyD88 knockout mice, the ability to resist enteric bacterial infection is severely compromised because of the combined negative effects of helminth infection and MyD88 deficiency. The current report advances our understanding of the MyD88-dependent signaling pathway in regulating intestinal mucosal immunity in host with multiple pathogen exposure.
